# Neural Correlates of Impaired Self-awareness of Deficits after Acquired Brain Injury: A Systematic Review

**DOI:** 10.1007/s11065-022-09535-6

**Published:** 2022-02-03

**Authors:** Anneke Terneusen, Ieke Winkens, Caroline van Heugten, Sven Stapert, Heidi I. L. Jacobs, Rudolf Ponds, Conny Quaedflieg

**Affiliations:** 1 Department of Neuropsychology and Psychopharmacology, Faculty of Psychology and Neuroscience, Maastricht University, Maastricht, the Netherlands; 2 Limburg Brain Injury Centre, Maastricht University, Maastricht, the Netherlands; 3 Department of Psychiatry and Neuropsychology, MHeNS School for Mental Health and Neuroscience, Maastricht University, Maastricht, the Netherlands; 4 Zuyderland Medical Centre, Department of Medical Psychology, Sittard-Geleen, the Netherlands

**Keywords:** Self-awareness, Unawareness, Acquired brain injury, Magnetic resonance imaging, Neural correlates

## Abstract

Self-awareness is essential for the process and outcome of rehabilitation but is often affected by acquired brain injury (ABI). While many studies investigated the psychological aspects of self-awareness deficits, the biological underpinnings are not well understood. The aim of this systematic review was to identify the neural correlates of self-awareness after ABI. Results indicate that anticipation of future problems is associated with lesions and decreased neural functioning in the right frontal lobe, as well as increased diffusivity throughout the white matter of the brain. Poor behavioral adjustment on implicit awareness tasks is associated with less functional connectivity of anterior cingulate cortex and right or middle inferior frontal gyri to the fronto-parietal control network, as well as more activation in the left insula and left parietal operculum during error processing. Recognition of mistakes is associated with internetwork connectivity of anterior or posterior default mode network to salience network. In conclusion, after ABI, different results in brain activation and connectivity are found depending on level of awareness measured. Future studies are necessary to confirm these findings.

**Supplementary Information:**

The online version contains supplementary material available at 10.1007/s11065-022-09535-6.

## Introduction

Acquired brain injury (ABI), such as stroke or traumatic brain injury (TBI), is one of the major causes of disability worldwide (GBD, 2015 Neurological Disorders Collaborator Group, 2017). People with severe ABI often suffer from a range of cognitive and behavioral deficits for which rehabilitation treatment is necessary (Azouvi et al., 2017). Unfortunately, the rehabilitation process is hindered when people exhibit reduced self-awareness of their deficits (Nowell et al., 2019). An impairment in self-awareness after ABI is associated with decreased motivation for and participation in therapy (Flashman & McAllister, 2002). Consequently, worse psychosocial rehabilitation outcomes are found, such as difficulties in societal participation, relationships, and return to work (Sherer et al., 1998). Therefore, self-awareness is an essential target of rehabilitation that requires attention, but is often considered a limiting factor in treatment success.

The exact cause of impaired self-awareness of deficits after ABI is unclear but most researchers and clinicians assume it is a combination of psychological factors and changes in brain functioning (Ownsworth et al., 2006). Despite that, literature to date has primarily focused on the psychological aspects, such as denial of changes in functioning, anxiety, defensiveness, and a variety of coping behaviors. Studies examining the neural correlates of self-awareness remain scarce, possibly due to the complexity of the topic or trouble recognizing it in clinical practice. However, understanding the neural aspects of self-awareness of deficits after ABI is necessary for the development of more comprehensive theoretical models of self-awareness. This, in turn, can contribute to the development of more effective measurement instruments, targeted interventions, and understanding limitations in current approaches of the problem.

One of the first influential models relating brain systems to self-awareness was presented by Stuss (1991) in which different types of self-awareness were categorized in a hierarchy. The lowest level of self-awareness is the actual knowledge and recognition of a deficit by the affected person. Lacking knowledge of a deficit often occurs in routinized behavior, such as speech. For example, patients with Wernicke’s aphasia (impairment of speech comprehension) might not be able to monitor and, thus, might not be aware of the nonsensical speech they produce. The next level is the integration of this knowledge into adaptive behavior. This executive control of novel or goal-directed behavior is regulated by the frontal lobes. Deficits at this level lead to impaired judgment or disorganized use of knowledge of a deficit. For example, one might be aware of memory problems but fail to adjust behavior accordingly, such as using a compensation strategy like a diary. At the highest level, the knowledge and executive control of a deficit is integrated into a timeframe. Memories and experiences of the past, perceptions and emotions of the present, and expectations of the future are used to create mental representations of the current self in the social environment. This is also regulated by the frontal lobes. With an impairment at this level of awareness, one might be unable to imagine that a deficit can lead to a problem in the future.

Since the introduction of this model, studies have consistently confirmed that behaviorally there are different types of self-awareness. Similar models have been created that isolate different aspects of self-awareness behavior. In general, this can be divided into online awareness of behavior during a specific task and offline awareness, which refers to reflecting on one’s behavior before or after a task (Brown et al., 2019). Similarly, efforts have been made to create optimal instruments to measure different self-awareness behaviors. Unfortunately, there is a gap in literature relating specific brain systems to these different levels of awareness, especially after ABI.

Empirical studies in non-ABI populations have confirmed the importance of the frontal lobes for self-awareness, but also suggest involvement of other brain regions from a neural network approach. For example, a study in individuals with neurodegenerative disease confirmed the role of frontal regions in cognitive self-awareness (Rosen et al., 2010). Similarly, the frontal lobes are of major importance for self-awareness in healthy individuals (Lou et al., 2017). However, studies indicate that other brain regions are involved in self-awareness in healthy participants as well, such as cortical midline structures (Lou et al., 2017; Northoff et al., 2006). Specifically, it has been suggested that the posterior cingulate cortex (PCC) is engaged when thinking about oneself and is influenced by the medial prefrontal cortex (mPFC; Davey et al., 2016). Over the years, several studies have been done on the neural correlates of self-awareness deficits after ABI as well, but there is no systematic overview of the findings yet.

There are various methods of assessment that can be used to differentiate between levels of self-awareness. A recent review classified the measurement instruments into three categories: (1) self-proxy discrepancy, (2) rating by clinician, and (3) performance-based discrepancy (Brown et al., 2019). In the self-proxy discrepancy method, patients’ self-ratings are compared to the rating of a significant other, caregiver, or therapist. This is often in questionnaire format and includes multiple domains. This requires judgments of one’s performance or deficits as well as integrating this knowledge into a timeframe and, therefore, reflects the highest level of self-awareness. The rating by a clinician is often based on interviews, observed behavior, and test scores. This could reflect the second level of awareness because clinicians can observe when errors are made and whether behavior is adapted accordingly. The performance-based methods are often basic tasks within a certain domain. Depending on the task, these can reflect all levels of self-awareness. The lowest level can be measured when, after a task, participants are explicitly asked whether mistakes were recognized, referred to in this paper as explicit self-awareness. If the task requires behavioral changes after an error has been recognized, this reflects the second level of self-awareness. These tasks rely on the assumption that self-awareness is necessary to successfully complete the task. Hence, these will be categorized as implicit self-awareness methods. Tasks that require mental representations of the self measure the highest level of self-awareness. Such tasks could include estimating expected performance prior to a task, anticipating problems in future situations, as well as retrospective reflection on behavior. By classifying the methods of assessment this way, the neural correlates at each level of self-awareness can be investigated.

The aim of this systematic review is to identify the structural and functional neural correlates of the different levels of self-awareness of cognitive and behavioral deficits after ABI. More specifically, associations between MRI-measures and the different measurement methods of self-awareness were investigated. The results are considered in relation to theoretical models of self-awareness after brain injury. This information leads to a better understanding self-awareness of deficits after ABI and could, ultimately, result in new or better approaches to treatment in the clinic.

## Methods

### Study Selection

This systematic review was designed according to the guidelines of Preferred Reporting Items for Systematic Review and Meta-Analysis Protocols (PRSIMA-P; Moher et al., 2015). Inclusion criteria were: (1) the study was published in English in a peer-reviewed journal, (2) the study was published during the 20 years before the search date, which was November 20th 2019, (3) the study population was human adults who were at least 18 years old and (4) were diagnosed with acquired brain injury such as stroke, TBI or hypoxia, (5) there was a direct measure of self-awareness, which could either be a self-proxy discrepancy score, performance-based discrepancy score, or a clinician-rated score, (6) structural or functional MRI were conducted as part of the study, and (7) the neuroimaging results relate certain brain areas or networks to self-awareness. Articles were excluded if they (1) included patients with neurodegenerative diseases, such as forms of dementia or Parkinson’s disease, or mixed samples with less than 50% ABI patients, (2) were single-case studies, (3) only measured self-awareness of physical or motor deficits, or (4) only retrieved imaging data retrospectively from medical files and not specifically performed scans as part of the study. The rationale for excluding studies concerning self-awareness of physical or motor deficits is that levels of self-awareness can differ per domain of functioning. For example, self-awareness of cognitive and behavioral deficits is often more impaired than that of physical deficits. Moreover, impaired self-awareness of motor deficits have been found to be unrelated to impaired self-awareness of cognitive deficits (Berti et al., 1996; Vocat et al., 2010). Given the complexity of impaired self-awareness and that the level of self-awareness can differ per domain, this paper focusses on self-awareness of cognitive and behavioral deficits, not that of physical deficits. The rationale for excluding scans derived from medical files was that the time between the initial scan, which is usually at emergency room admittance, and the self-awareness measurement for the study could be years, and brain structure and function could have changed in the meantime.

The databases Pubmed, PsycINFO, Web of Science and EMBASE were systematically searched for relevant literature on November 20th 2019. The full list of search terms can be found in Appendix 1. Search terms were always terms relating to self-awareness combined with terms relating to imaging techniques and terms relating to brain injury. Titles of all potentially relevant studies were collected in Endnote X8.2. Duplicates were removed. After inspection of inclusion and exclusion criteria, titles were screened for eligibility by one author (AT). If there was doubt, the study was included. Relevant abstracts were uploaded and screened for eligibility in Covidence by two authors (AT and CQ). Any disagreements were discussed and resolved. If inclusion or exclusion was unclear based on the abstract, full texts were assessed. Reference lists of included articles were checked for further relevant articles.

### Classification, Data Collection, and Synthesis

The included studies were first classified according to method of self-awareness assessment, including self-proxy discrepancy, rating by clinician, or performance-based discrepancy (Brown et al., 2019). A second classification was made based on imaging technique. Structural imaging studies were separated from functional imaging studies.

Variables extracted from the studies included demographical factors such as sample size, age, sex, education, time since injury, type of injury, and injury severity. Data gathered on self-awareness assessment included measurement technique, awareness score, and interpretation of that score. Additionally, information on imaging procedure, imaging technique, and task contrast was extracted. If relevant, the task used during imaging was noted. The imaging results and interpretations of which brain areas are involved in self-awareness were also extracted from the papers. The relevant data was extracted from the studies by two authors independently (AT and CQ). This was then compared and any discrepancies were resolved together by referring back to the paper. The principal measures for this review were results relating self-awareness of deficits to structural and functional neural correlates. Brain areas involved in self-awareness of deficits and directions of correlations were summarized and interpreted in relation to methodological differences such as type of self-awareness and imaging technique.

### Quality Assessment

Quality of the included studies was assessed by two authors (AT and CQ; see Fig. 2). Quality of the studies was evaluated based on the nine criteria described in Wolters et al. (2019): (1) description of participants, (2a) description of imaging procedure and instructions, (2b) description of self-awareness measure, (3) description of imaging analysis, (4) specification of regions of interest, (5) reproducibility, (6) statistical testing, (7) multiple testing problem, (8) figures and tables, and (9) quality control measures. Criteria 2a and 2b were specified in the current study to ensure that the self-awareness measure as well as the imaging procedure were well described. Each criterion could be scored with 1 point (+), 0.5 points (±), or 0 points (-). Total score was calculated and corrected for number of applicable criteria (total score or number of applicable criteria times 10). A score of 7.5 or higher was considered as good quality, a score between 4 and 7.5 as fair quality, and a score of 4 or less as poor quality (Wolters et al., 2019). Quality of papers is considered while interpreting and discussing the results of the data synthesis. The full assessment tables per study can be found in the Supplementary material.

## Results

### Search Results and Classification

**Fig. 1:**
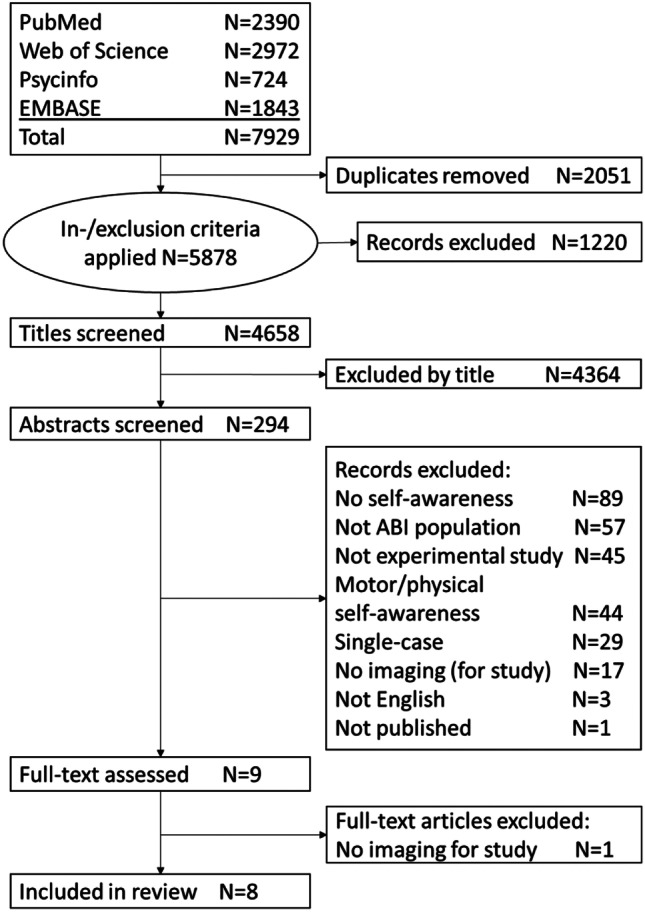
Flowchart of selection procedure

The flowchart of the selection procedure can be found in Fig. 1. After title screening (N = 4658) and abstract screening (N = 294), nine full texts were assessed of which eight were included in the review. Three studies used self-proxy discrepancy scores to measure self-awareness (Bivona et al., 2014; Lesimple et al., 2019; Tezuka et al., 2013), three studies used performance-based discrepancy scores (Garcia-Cordero et al., 2019; Grossner et al., 2018, 2019), and two studies included both self-proxy discrepancy scores as well as performance-based discrepancy scores (Ham et al., 2014; Schmitz et al., 2006). There were no studies in which a rating by a clinician was used to measure self-awareness. Study characteristics can be found in Table 1.

### Self-proxy Discrepancy Method

**Fig. 2:**
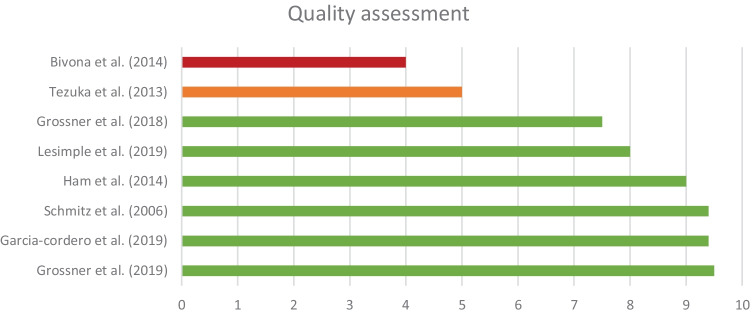
Quality assessment scores per study. Based on nine criteria described in Wolters et al. (2019). Each criterion could be scored with 1 point (+), 0.5 points (±) or 0 points (-). Total score was calculated based on number of applicable criteria. A score of 7.5 or higher was considered as good quality (green), 4–7.5 as fair quality (orange) and 4 or less as poor quality (red)

As can be seen in Fig. 2, one paper that used self-proxy discrepancy scores to measure self-awareness was of poor quality (Bivona et al., 2014), one of fair quality (Tezuka et al., 2013), and three papers were of good quality (Ham et al., 2014; Lesimple et al., 2019; Schmitz et al., 2006). The self-proxy discrepancy score in the study by Ham et al. (2014) was merely used to confirm self-awareness levels of the groups. These results were not directly associated to neuroimaging findings and, thus, will not be further discussed in this section.

All four studies used different questionnaires to measure self-awareness, including the Barthel Index (Tezuka et al., 2013), Awareness Questionnaire (Bivona et al., 2014), Dysexecutive Questionnaire (Lesimple et al., 2019), and Patient Competency Rating Scale (Schmitz et al., 2006). In these questionnaires, people estimate how well or poorly they will be able to perform specific daily life activities in the near future and, thus, require mental representations of the self in the environment. The proxies who filled in the questionnaires were either patients’ primary caregivers (Tezuka et al., 2013) or relatives (Bivona et al., 2014; Lesimple et al., 2019; Schmitz et al., 2006). Self-awareness scores were calculated as the difference in total score between the patient and the primary caregiver or relative. Higher discrepancy scores represent poorer self-awareness. Behavioral results showed that certain ABI groups overestimated their functioning (Schmitz et al., 2006; Tezuka et al., 2013) and underestimated their deficits (Lesimple et al., 2019).

#### Structural Neural Correlates of Self-Awareness Based on Self-proxy Discrepancies

**Fig. 3:**
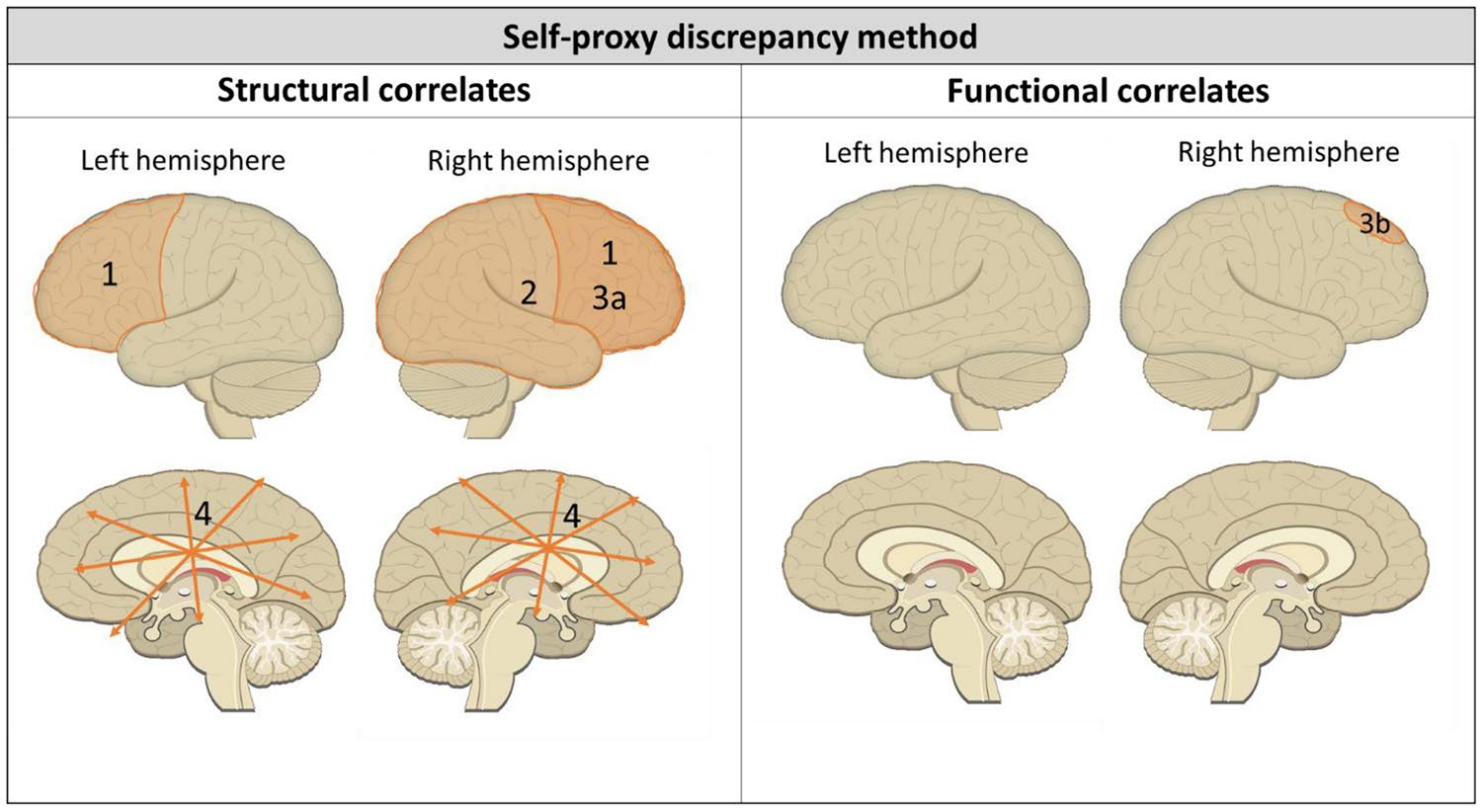
Structural and functional neural correlates of impaired self-awareness as measured with self-proxy discrepancy scores after ABI. 1. Lesions in frontal cortical areas associated with impaired self-awareness (Bivona et al., 2014). 2. Right hemisphere lesions associated with impaired self-awareness (Tezuka et al., 2013). 3a. Right frontal lobe gray and white matter damage associated with impaired self-awareness. 3b. Less BOLD activation in right superior frontal gyrus during self-evaluation task associated with impaired self-awareness (Schmitz et al., 2006). 4. Higher mean diffusivity in the whole white matter associated with impaired self-awareness (Lesimple et al., 2019)

Structural MRI or CT scans were used to determine lesion location (Bivona et al., 2014; Tezuka et al., 2013), injury severity (Schmitz et al., 2006), and white matter abnormalities (Lesimple et al., 2019). Results are depicted in Fig. 3. When comparing TBI patients with impaired self-awareness to patients with adequate self-awareness, the distribution of lesions showed that those with impaired self-awareness had significantly more frontal lesions (shaded area 1 in Fig. 3; Chi-square = 8.97; *p* < 0.01), but there were no differences with regard to diffuse or posterior cortical lesions (Bivona et al., 2014). Comparing patients with right to left hemisphere cerebrovascular lesions, it was found that the self-proxy discrepancy score was significantly larger in those with right hemisphere lesions (shaded area 2 in Fig. 3; Mann–Whitney test; *p* < 0.0001), indicating more impaired self-awareness (Tezuka et al., 2013). The other two studies correlated patients’ structural neuroimaging findings to the self-proxy discrepancy scores on the self-awareness measures. An almost significant positive correlation (*r* = 0.44; *p* = 0.055) was found between impaired self-awareness and right frontal lobe gray and white matter damage (shaded area 3a in Fig. 3), whereas this correlation was much weaker (*r* = 0.29; *p* = 0.22) in TBI patients with left frontal lobe damage (Schmitz et al., 2006). Furthermore, mean diffusivity in the whole white matter of TBI patients positively correlated with impaired self-awareness (area 4 in Fig. 3; *r* = 0.26; *p* = not reported; Lesimple et al., 2019).

#### Functional Neural Correlates of Self-awareness Based on Self-proxy Discrepancies

One study used the self-proxy discrepancy score to predict brain activation during a self-evaluation task (Schmitz et al., 2006). In the task, TBI patients had to rate trait adjectives as relating to themselves or objectively rate whether the valence of the trait adjective was positive or not. Brain activity was compared between these two conditions, and the difference was attributed to self-awareness. Subsequently, it was investigated whether patient competency rating scale (PCRS) discrepancy scores were predictive of the self-awareness-related brain activation seen during the task. Higher self-proxy discrepancy scores (indicating less self-awareness) were associated with less BOLD signal difference between conditions in the right superior frontal gyrus (SFG) during the self-awareness task (shaded area 3b in Fig. 3; *r* = -0.26; *p* = 0.005; Schmitz et al., 2006). This correlation was not significant in the left SFG (*r* = -0.23; *p* = 0.16; Schmitz et al., 2006).

#### Summary Self-proxy Discrepancy Method

The four studies above suggest that impaired self-awareness after ABI, as measured by self-proxy discrepancy scores, is associated with lesions, white and gray matter damage, and decreased task-related activation in the right frontal lobe, as well as higher mean diffusivity in the whole white matter. In the study by Tezuka et al. (2013), participants were stroke patients, there was a larger proportion female participants than male participants, mean age was around 80 years old, and injury severity was not reported. The three other studies included TBI patients, had a larger proportion of male participants, mean age ranged from 27.1 to 39.7 years old, and injury severities were moderate to severe. In light of these differences, the results reported by Tezuka et al. (2013) might be less comparable to the others. Results of Bivona et al. (2014) and Lesimple et al. (2019) should be interpreted with caution since they did not correct for multiple testing. Nevertheless, the overall conclusion remains that right frontal brain areas and white matter diffusivity throughout the brain are involved in impaired self-awareness after ABI when measured by self-proxy discrepancy scores.

### Performance-based Discrepancy Method

All of the studies measuring self-awareness by means of a performance-based discrepancy method were of good quality, as can be seen in Fig. 2 (Garcia-Cordero et al., 2019; Grossner et al., 2018, 2019; Ham et al., 2014; Schmitz et al., 2006). For the purpose of this review, the methods are split into explicit self-awareness methods and implicit self-awareness methods. In explicit self-awareness methods, the participants were consciously triggered to evaluate their performance, while in implicit self-awareness methods they were not.

#### **Implicit Self-awareness**

Using a simple perceptual task in which participants had to select the largest circle on the screen, Garcia-Cordero et al. (2019) asked stroke patients whether they wanted to stick with their answer and risk winning or losing three points, or go for the safe option to opt out and receive one point. Self-awareness in this task is related to the second level of awareness, adapting behavior. Namely, implicit performance monitoring was the fraction of times participants would take the risk out of the total number of these types of trials. Stroke patients showed significantly less implicit monitoring than healthy controls in this risk-taking task (Garcia-Cordero et al., 2019). Another study assessed implicit self-awareness during a stop-signal task, which allows dissociation of error processing and response inhibition (Ham et al., 2014). This task also measures self-awareness on the level of behavioral change. Accuracy on the stop-signal task was the same when comparing an impaired self-awareness TBI group to an adequate self-awareness TBI group and healthy control group on the stop-signal task (Ham et al., 2014). However, the impaired self-awareness group was generally slower to respond, had greater intra-individual variability, and showed greater post-error slowing (Ham et al., 2014).

#### Explicit Self-awareness

Explicit self-awareness was assessed using behavioral tasks in combination with a confidence judgment after each trial. These tasks require recognition of errors, reflecting the first level of awareness. In the perceptual task used by Garcia-Cordero et al. (2019), participants were asked to report confidence of their answer on a slider ranging from low confidence to high confidence. The mean of these values was used as a measure of explicit monitoring. Stroke patients did not significantly differ from controls regarding confidence (Garcia-Cordero et al., 2019). Another study, described in two papers, used an adapted version of the Matrix reasoning task in which participants had to select an image to complete a pattern. After each trial, they were asked to rate how confident they were of their answer, ranging from completely certain to completely uncertain on a 6-point Likert scale (Grossner et al., 2018, 2019). Self-awareness was measured as metacognitive accuracy and calculated using the area under a receiver operating characteristic (AUROC) curve. Behavioral results indicate that TBI patients did not differ from healthy controls in levels of explicit self-awareness (Grossner et al., 2018). Finally, Schmitz et al. (2006) used a task in which participants had to rate trait adjectives as relating to themselves or objectively rate whether the valence of the trait adjective was positive or not. Brain activity was compared between these two conditions, and the difference was attributed to self-awareness. This task requires mental representations of the self, reflecting the highest level of awareness. Results show no significant behavioral difference between TBI patients and healthy controls (Schmitz et al., 2006).

#### Structural Neural Correlates of Self-Awareness Based on Performance-Based Discrepancies

##### Implicit Self-awareness

**Fig. 4:**
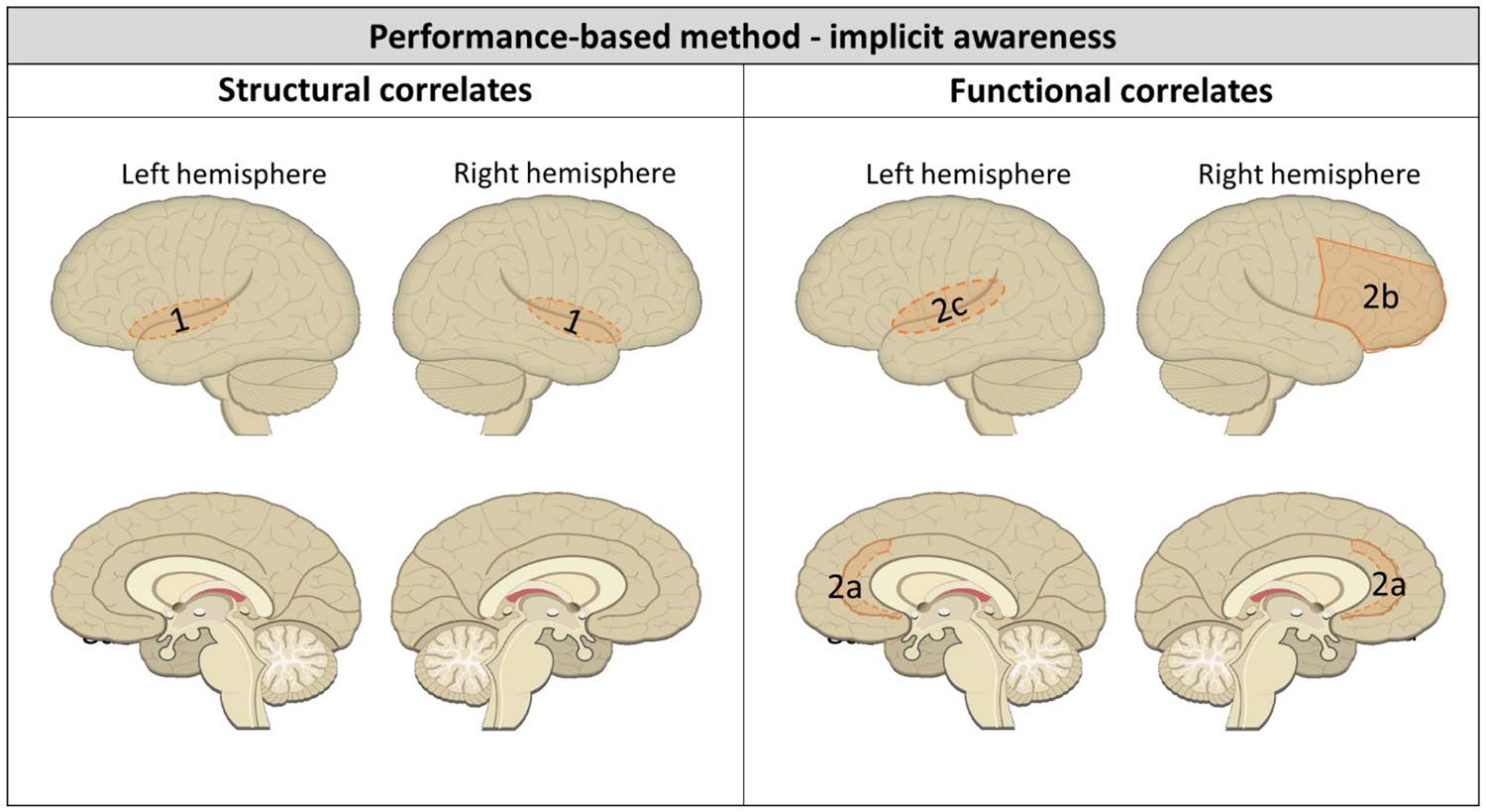
Structural and functional neural correlates of impaired implicit self-awareness as measured with performance-based discrepancy scores after ABI. 1. Fronto-temporo-insular lesions associated with impaired implicit self-awareness (Garcia-cordero et al., 2019). 2. Less functional connectivity of the 2a. dorsal anterior cingulate cortex and 2b. right middle and inferior frontal gyri to the rest of the FPCN in resting state associated with impaired implicit self-awareness. 2c. Increased activation in left insula and left parietal operculum during error processing (stop-signal task) associated with impaired implicit self-awareness (Ham et al., 2014)

Results are visualized in Fig. 4. Voxel-based lesion symptom mapping was used to identify which lesion locations correlated with implicit and explicit awareness in stroke patients (Garcia-Cordero et al., 2019). For implicit self-awareness, a positive association was found between lesions in fronto-temporo-insular brain areas and impaired implicit awareness (shaded area 1 in Fig. 4; t-score > 1.82, *p* < 0.05, FDR-corrected; Garcia-Cordero et al., 2019). Another study compared focal lesion location and volume between adequate and impaired TBI patient groups using lesion overlap images. However, formal statistical analysis was not possible due to a lack of common lesions, indicating no obvious relationship (Ham et al., 2014). In the same study structural connectivity of the dorsal anterior cingulate cortex (ACC) to bilateral insulae (tracts of fronto-parietal control network; FPCN) was assessed using diffusion tensor imaging (DTI), comparing a group of TBI patients with impaired self-awareness to a group of TBI patients with adequate self-awareness and a control group (Ham et al., 2014). Likewise, no significant differences were found between the brain-injured groups regarding fractional anisotropy values in this tract (Ham et al., 2014).

##### Explicit Self-awareness

**Fig. 5:**
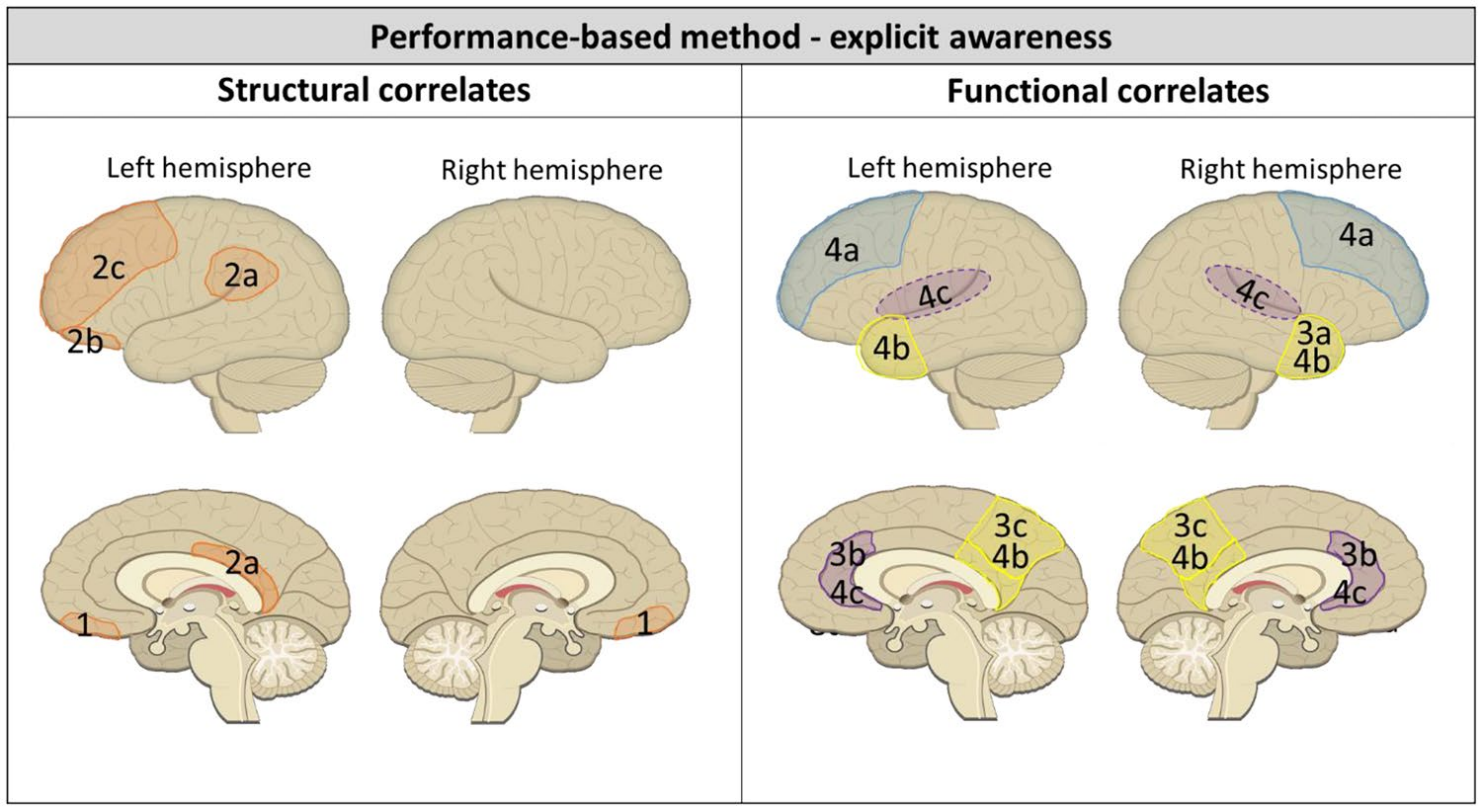
Structural and functional neural correlates of impaired explicit self-awareness as measured with performance-based discrepancy scores after ABI. 1. Ventromedial frontal lesions associated with explicit self-awareness (Garcia-cordero et al., 2019). 2. Lack of positive association, that is present in healthy controls, between explicit self-awareness and gray matter volume in the: 2a. left posterior region (posterior dorsal and ventral parts of the cingulate cortex, angular gyrus and supramarginal gyrus); 2b. left orbital region (orbital gyrus and orbital H-shaped sulcus); 2c. left dorsolateral region (superior & middle frontal gyrus, middle frontal sulcus) in TBI patients (Grossner et al., 2018). 3. During self-related task, increased activation in 3a. right anterior temporal pole, 3b. anterior cingulate cortex, and 3c. precuneus in TBI compared to healthy controls (Schmitz et al., 2006). 4. Explicit self-awareness is positively associated with internetwork connectivity between 4a. anterior default mode network (blue; middle and superior frontal regions) and 4c. salience network (purple; insular cortex and anterior cingulate); and between 4b. posterior default mode network (yellow; posterior cingulate, precuneus and temporal pole) and 4c. salience network (purple; insular cortex and anterior cingulate) in TBI patients (opposite associations for controls) (Grossner et al., 2019)

A depiction of the results can be found in Fig. 5. Voxel-based lesion symptom mapping showed positive associations between ventromedial lesions and confidence (shaded area 1 in Fig. 5; t-score > 2.88, *p* < 0.05, FDR-corrected), which was used as measure of explicit self-awareness (Garcia-Cordero et al., 2019). Another study showed that in a healthy control group, better explicit self-awareness was associated with more gray matter volume in the left posterior region (shaded area 2a in Fig. 5; PCC, angular gyrus, and supramarginal gyrus), the left orbital region (shaded area 2b in Fig. 5; orbital gyrus and orbital H-shaped sulcus), and the left dorsolateral region (shaded area 2c in Fig. 5; superior frontal gyrus, middle frontal gyrus, and middle frontal sulcus; Grossner et al., 2018). Remarkably, these associations were absent in the brain-injured population, where self-awareness was not significantly associated with total or regional gray matter volume (Grossner et al., 2018). However, total gray matter volume was lower in the TBI group. Moreover, gray matter volume in the right hemisphere was not significantly associated with self-awareness in either group in this study (Grossner et al., 2018).

#### Functional Neural Correlates of Self-awareness Based on Performance-based Discrepancies

##### Implicit Self-awareness

A summary of the results can be found in Fig. 4. Functional connectivity within the FPCN was compared between a group of TBI patients with impaired self-awareness and one with adequate self-awareness (Ham et al., 2014). The FPCN in this study included dorsal ACC, bilateral insulae, bilateral temporo-parietal junctions, and bilateral middle and inferior frontal gyri. Results show that in resting state, the impaired self-awareness TBI group showed significantly less functional connectivity of the dorsal ACC (shaded area 2a in Fig. 4), right inferior frontal gyrus and right middle frontal gyrus (shaded area 2b in Fig. 4) to the rest of the FPCN (Ham et al., 2014). Additionally, brain activation during a stop-signal task was compared between these groups. During error processing, the impaired self-awareness TBI group showed increased activation in the left insula and left parietal operculum (shaded area 2c in Fig. 4; Ham et al., 2014). Brain activation after successful response inhibition was similar in adequate and impaired self-awareness groups (Ham et al., 2014).

##### Explicit Self-awareness

Results are shown in Fig. 5. During a self-evaluation task, in which participants were relating adjectives to themselves as opposed to objectively rating their valence, TBI patients and healthy controls both showed activation in midline cortical structures (Schmitz et al., 2006). However, compared to healthy controls the TBI group showed significantly more activation in the right anterior temporal pole (shaded area 3a in Fig. 5; maxima *t* = 4.12, *df* = 19, *p* uncorrected < 0.001; Schmitz et al., 2006), ACC (shaded area 3b in Fig. 5; maxima *t* = 4.72, *df* = 19, *p* uncorrected < 0.001), and precuneus (shaded area 3c in Fig. 5; maxima *t* = 3.68, *df* = 9, *p* uncorrected < 0.001). In another study, resting state functional connectivity between and within brain networks including the attention network, default mode network (DMN), salience network, and frontoparietal network were compared between a TBI group and a healthy control group (Grossner et al., 2019). Grossner et al. (2019) report significant interactions between group and internetwork connectivity of anterior DMN to salience network on explicit self-awareness (*R*^*2*^ = 0.13, *p* = 0.047). Furthermore, they report significant interactions between group and internetwork connectivity of posterior DMN to salience network on explicit self-awareness (*R*^*2*^ = 0.15, *p* = 0.038). Specifically, in the TBI group, there were positive relationships between internetwork connectivity and explicit self-awareness, while in the control group these relationships were negative. The anterior DNM included middle and superior frontal regions (shaded area 4a in Fig. 5); the salience network included the insular cortex and ACC (shaded area 4c in Fig. 5); the posterior DMN included PCC, precuneus, and temporal poles (shaded area 4b in Fig. 5; Grossner et al., 2019).

#### Summary Performance-based Discrepancy Method

Five good quality studies investigated neural correlates of impaired self-awareness as measured by performance-based discrepancy scores. Regarding demographics, four studies were rather similar and included populations of moderate to severe TBI patients with a larger proportion of males than females. One study population stood out from the rest with a slightly larger proportion of females, mean age of 61.9 years, and participants had suffered a fronto-insular stroke (Garcia-Cordero et al., 2019). This study might be less comparable to the others. Results of Ham et al. (2014) and Grossner et al. (2019) should be interpreted with caution since they did not correct for multiple testing. Behavioral results concerning implicit self-awareness are mixed, and so are the structural imaging results. Brain areas functionally associated with implicit self-awareness after ABI included the ACC, frontal gyrus, left insula, and parietal operculum. These areas overlap with the networks of which internetwork connectivity is associated with explicit self-awareness. While no behavioral discrepancies between ABI and control groups were found on explicit self-awareness tasks, the imaging results do indicate differences. Again, structural results are mixed. Functional imaging results indicate more BOLD response in ABI patients compared to controls in the ACC, precuneus, and right anterior temporal pole during a self-related task. Furthermore, the association between explicit self-awareness and internetwork connectivity of anterior or posterior DMN to salience network in ABI patients is the opposite of that in healthy controls.

## Discussion

Self-awareness is crucial for rehabilitation outcome but often impaired after ABI. Understanding the underlying neural correlates of self-awareness of cognitive, emotional, and behavioral deficits after ABI is important for theoretical comprehension as well as the development of measurement instruments and interventions. Therefore, the literature on structural and functional neural correlates of self-awareness was systematically reviewed. Eight studies of poor to good quality were included and results were reported in relation to the different measurement methods and levels of self-awareness. A distinction was made between self-proxy discrepancy scores and performance-based self-awareness scores.

Overall, the current review gives insight into the neural consequences of ABI on self-awareness. Individuals who suffered an ABI show altered functioning and connectivity in brain networks that are associated with self-awareness. The results confirm that the frontal lobes are associated with changed and often impaired self-awareness after ABI, as suggested by (Stuss, 1991). Moreover, they propose a network approach in which other regions are important too. Namely, a neural network of frontal and cortical midline regions corresponding to the anterior DMN (including middle and superior frontal regions), posterior DMN (including PCC, precuneus and temporal poles), and the salience network (including the insular cortex and ACC).

### Theoretical Integration

Self-awareness of deficits is theoretically complex and there is a variety of terms to describe the construct. They all refer to a mismatch between an objective and subjective perceived level of functioning. As described in Brown et al. (2019), most models of self-awareness make a distinction between online and offline awareness. Online awareness refers to awareness of one’s performance during a task, while offline awareness refers to reflecting on one’s behavior before or after a task. Measuring self-awareness using self-proxy discrepancy scores on a questionnaire including multiple domains requires judgments of one’s deficits as well as integration of this knowledge into a timeframe. Therefore, this reflects offline awareness. In the model described by Stuss (1991), this would be the highest level of awareness. In the pyramid model by Crosson et al. (1989) this would entail anticipatory awareness, which is comparable to metacognitive knowledge in the model described by Toglia and Kirk (2000). The current review demonstrates that an impairment in this type of self-awareness is associated with lesions and decreased neural functioning in the right frontal lobe, as well as increased diffusivity throughout the white matter of the brain. One performance-based study investigated self-related brain activity, which fits in this highest level of self-awareness too. TBI patients showed over-activation in ACC, precuneus, and right anterior temporal pole during the self-related task compared to healthy controls.

Measuring online self-awareness as a performance-based discrepancy can reflect either the second or the lowest level in the model described by Stuss (1991). The implicit performance-based studies, in which participants had to show adaptive goal-directed behavior during a task, reflect the second level of self-awareness. This corresponds to emergent awareness in the pyramid model (Crosson et al., 1989), and online awareness in Toglia and Kirk’s (2000) model. An impairment on this level is associated with less functional connectivity of the ACC and right and middle inferior frontal gyri to the FPCN, as well as more activation in the left insula and left parietal operculum during error processing. The lowest level of self-awareness (knowledge of an impairment or recognition of mistakes made in tasks), reflects intellectual awareness, or a phase in between intellectual and emergent awareness, in the pyramid model (Crosson et al., 1989) and part of metacognitive knowledge in the model described by Toglia and Kirk (2000). This was associated with internetwork connectivity of anterior or posterior DMN to salience network. Specifically, in TBI patients more internetwork connectivity at rest was associated with better self-awareness.

### Comparison to Healthy Individuals and Other Patient Populations

Our findings are in line with previous research in healthy populations in which reflecting on the self, the highest level of self-awareness, was associated with cortical midline structures (Lou et al., 2017; Northoff et al., 2006) and the DMN (Davey et al., 2016). Anterior components of the DMN are suggested to be involved in perception and judgement, while the posterior components are important for episodic memory retrieval (Lou et al., 2004; Uddin et al., 2009). More specifically, it has been suggested that the PCC regulates self-related processes and is influenced by the mPFC, which can gate this information into conscious awareness by weighing internal and external demands (Davey et al., 2016). Furthermore, the connection between DMN and salience network is involved in cognitive control (Scheibel, 2017). We found that after ABI, this internetwork connectivity is associated with better self-awareness. The salience network consists of the ACC and insula. The ACC is associated with performance monitoring. It can detect errors, assess task performance, and signal for behavioral change (Scheibel, 2017). The results in this systematic review suggest that after ABI, the ACC is less functionally connected to the FPCN and that this is associated with poor self-awareness in terms of error-correction (second level of awareness). The ACC also showed more BOLD activation during a self-reflection awareness task (highest level of awareness) in the ABI population compared to healthy controls, while behavioral results were similar. These results suggest a role of the ACC in self-awareness deficits after ABI.

Individuals diagnosed with Alzheimer’s disease (AD) also frequently show impaired self-awareness. The DMN is known to be affected in AD (Buckner et al., 2008). In line with our results, a recent review concluded that reduced functional connectivity within the DMN in mild to moderate AD is associated with impaired self-awareness measured by questionnaires or self-report (Mondragón et al., 2019). Furthermore, in the initial stages of cognitive decline in AD, impaired self-awareness is associated with neural dysfunction in cortical midline structures, including mPFC, ACC, and PCC. This later progresses to the parietotemporal structures, and, ultimately, to frontotemporal dysfunction (Mondragón et al., 2019). Brain damage after ABI is not neurodegenerative like in AD. Nevertheless, the brain areas found in later stages of AD correspond with these results that show involvement of midline as well as parietotemporal and frontal structures in self-awareness after ABI. However, how these areas influence each other and the generation of awareness remains unclear.

### Considerations and Future Directions

Different measures of self-awareness were used in the studies included in this systematic review. On a behavioral level, the self-proxy discrepancy methods showed significant differences between groups, while performance-based methods often did not. This could be explained by the fact that they measure different levels of awareness. It is important to have these various instruments to be able to measure all aspects of self-awareness. However, it is difficult to compare results within one level of awareness when different instruments are used to measure the same aspect of awareness. The self-proxy discrepancy methods are comparable in that they all compare a patient’s own estimation to that of a primary caregiver or relative. However, these questionnaires are somewhat different in what they measure. Some measure estimations of performance, while others measure estimations of impairments. Some focus solely on post-injury status, while others compare pre-injury to post-injury status. Therefore, it is important to consider which questionnaires to use at which time points when studying the variance in self-awareness deficits. Nevertheless, they all measure distinct aspects of the highest level of self-awareness and, as such, all contribute to the understanding of self-awareness.

For the performance-based methods, two types of awareness can be measured including recognition of mistakes and adjustment of one’s behavior. In the latter, it is assumed that proper self-awareness is necessary for adaptive goal-directed behavior. However, it is important to realize that some of these implicit awareness tasks might have measured other behaviors such as risk-taking or inhibition. While these factors are linked to self-awareness, these behaviors are not equivalent to self-awareness. In the explicit performance-based methods, which measure recognition of mistakes, participants had to rate how confident they were of their answer by indicating this on a slider or scale. This was not always compared to actual performance and, therefore, it is not always clear whether there was under-confidence, appropriate confidence, or over-confidence. This can be overcome by using a type II area under the receiver operating characteristic curve (AUROC) as was done in Grossner et al. (2018) and Grossner et al. (2019). This is a sophisticated method that combines one’s accuracy on the task with one’s subjective confidence rating to create a performance-based self-awareness score (Fleming & Lau, 2014), and should be considered in future research. Another interesting paradigm to consider in future studies is the judgment of learning paradigm in which participants are asked to estimate how well they expect to perform before doing a task (Nelson, 1990). This was not done in the current studies but might be very relevant since it is part of metacognitive strategy training (Kennedy et al., 2008) which, in turn, is one of the therapies known to improve rehabilitation outcomes in ABI patients with impaired self-awareness (Engel et al., 2019).

A first limitation of this review is that the number of studies explicitly investigating neural correlates of self-awareness after ABI is limited. A meta-analysis of the imaging results was not feasible due to the large variety in awareness measurements, imaging techniques, and study designs. The limited number of studies on this topic could be due to the inherent complexity of recognizing self-awareness deficits and quantifying them. Research has primarily focused on the psychological and behavioral aspects of impaired self-awareness. However, the developments in neuroscience tools over the past years, such as neuroimaging, provide opportunities to bridge this gap in literature and gain further understanding of the neural aspects of self-awareness after ABI. Another consideration to keep in mind is the mixed etiology of acquired brain injury. Studies on both TBI and stroke patients were included in this review. The majority of the studies included in this review were on TBI patients. Therefore, the current results are most relevant to that population. Nevertheless, especially given the low number of studies on this topic, reviewing self-awareness in ABI is a good starting point. Further research could look into the differences between etiologies within ABI, as it might be possible that impaired self-awareness evolves differently in different etiologies. Thirdly, it is important to note that some studies applied multiple imaging techniques or self-awareness measurement methods on the same, or parts of the same, population. Therefore, some samples come back several times in this systematic review. It is necessary to conduct more studies with different participants so that results are more generalizable. Some of these studies did not correct for multiple testing. Although this was considered in the quality assessment, these results must be interpreted with caution. Lastly, studies on self-awareness of physical or motor deficits were excluded in the current systematic review. While this may limit the generalizability, this approach increases the specificity of our results. Self-awareness of cognitive and behavioral deficits are found to be unrelated to self-awareness of physical deficits (e.g. Vocat et al., 2010). Given the complexity of impaired self-awareness and that the level of self-awareness can differ per domain, focusing on the cognitive domain increases the specificity of the results and, hence, the clinical utility.

The current review indicates that brain areas within the salience network and the DMN are involved in self-awareness. Activity and functional connectivity in resting state as well as during tasks are affected after ABI and should be further examined. There is a need for studies using multiple imaging techniques and a combination of self-awareness measurement methods to gain insight into different types of self-awareness within the same population. These studies can provide useful information that can be used to compare self-awareness after ABI to other populations, verify theoretical models, and improve interventions. For example, a recent development in the literature is combining targeted brain stimulation with cognitive rehabilitation. Brain stimulation can enhance brain plasticity and, in combination with cognitive therapy, create a synergistic effect that enhances the effectiveness of rehabilitation (Miniussi & Rossini, 2011; Sathappan et al., 2019). This could be relevant for self-awareness since network functioning appears to be disturbed and, if that can be manipulated, this could aid rehabilitation.

In conclusion, knowledge of impairments, controlling behavior accordingly, and future anticipation of the self in specific situations are distinct aspects of self-awareness that unite in adaptive behavior. Different measurement methods and MRI techniques have been used to assess the neural correlates of self-awareness at each level. Overall, areas of the DMN and salience network were consistently found to be involved in self-awareness after ABI. More research is needed to confirm these findings and further investigate the role of these brain areas in the different levels of self-awareness deficits after ABI.

**Table 1:** Characteristics of studies measuring neural correlates of self-awareness of cognitive deficits in acquired brain injury

**Study Year**	**Study (year)**		**♂/♀**		**Age in years mean (SD)**		**Injury** **Time**^**a**^	**Injury severity**^**b**^		**Self-awareness**				Imaging
	ABI	HCN	ABI	HC	ABI	HC	mean (SD)		**Method**^**c**^	**Measure**	**Finding**	**Technique**	**Contrast**	**Significant findings**
Tezuka et al. (2013)	31 LHD stroke		10/20		79.1 (8.5)		≥ 3			BI patient relative	Overestimation of activities daily living in RHD	Structural: CT or MRI	Left hemisphere damage (LHD) vs. Right hemisphere damage (RHD)	RHD associated with impaired self-awareness
	30 RHD stroke		10/21		80.7 (8.8)		≥ 3							
Bivona et al. (2014)	14 TBI-ISA	28	12/2	21/7	37.2 (13.3)	34.5 (9.9)	27.3 (25.4)^d^	S	S-P	AQ patient - relative	Median split to make groups	Structural: CT or MRI	Impaired (ISA) vs adequate (ASA) self-awareness	Damage in frontal cortical regions ISA > ASA No difference in axonal injury or posterior cortical regions
	14 TBIASA		9/5		30.6 (8.9)		25.7 (20.6)^d^							
Lesimple et al. (2019)	63 TBI		55/8		39.7 (15.0)		63.4 (20.7)	S	S-P	DEX relative -patien	Underestimation of deficits	Structural: MRI, DWI	Within TBI: correla-tions white matter measures and self-awareness	Diffusivity in whole white matter negatively associated with self-awareness
Schmitz et al. (2006)	20 TBI	20	13/7	8/12	27.1 (10.8)	28.1 (11.4)	2.7 (0.6)^d^	M	S-P	PCRS patient - relative	Overestimation of performance	Structural: MRI	Left frontal (LFD) vs right frontal (RFD) damage correlations with self-awareness	RFD positively correlated with impaired self-awareness
									PB	Self-evaluation task	Equal performance for TBI and HC	Functional: task fMRI	TBI vs HC: activation during self-evaluation task vs word valence task: within + be-tween groups. Within TBI: self-evaluation activation regressed with pre-dictor PCRS discrep-ancy	TBI > HC: anterior cingulate cortex, precuneus and right anterior tem-poral pole Right superior frontal gyrus func-tioning associated with self-awareness
Ham et al. (2014)	63 TBI	24	46/17	16/8	38.0 (12)	36.2 (10.2)	29 (74)		S-P	FrSBe patient - relative	Underestimation of deficits in ISA	Structural: MRI, DTI	ISA vs ASA vs HC: measures of white matter within fronto-parietal control net-work (FPCN)	No differences
	23 ISA		13/10		39.4 (12.8)		46.9 (115.3)	M/S						
	40 ASA		33/7		37.3 (11.6)		19.7 (30.1)							
Subset	48 TBI	25	37/11	17/7	35.7(10.9)	34.8 (9.6)			PB	Stop-change task	Lower performance monitoring in ISA	Functional: rest Fmri task-fMRI	ISA vs ASA vs HC: functional connectivi-ty whole brain + ROI nodes of FPCN ISA vs ASA vs HC: functional connectivi-ty during error pro-cessing + response inhibition	ISA reduced connectivity FPCN to dorsal anterior cingulate cortex, right middle frontal gyrus and right inferior frontal gyrus ISA reduced connectivity FPCN to dorsal anterior cingulate corteISA > ASA: left insula and left parie-tal operculum x, right middle frontal gyrus and right inferior frontal gyrus
	18 ISA		NR		NR		NR							
	30 ASA		NR		NR		NR							
Grossner et al. (2018)	34. TBI	28	19/15	16/12	33.4 (13.1)	34.1 (12.6)	51.7 (85)	S	PB	Reasoning task	Equal performance for TBI and HC	Structural: MRI	TBI vs HC: gray matter volume whole brain + ROI frontal, temporal and occipital regions; and correlations with self-awareness	HC positive association between metacognitive accuracy and left posterior, left orbital and left dorso-lateral regions; TBI no association
Grossner et al. (2019) Partly overslaps with 2018 study	21-TBI	23	12/9	12/11	32.9(14.0)	36.9 (12.6)	49.9 (77.0)	S	PB	Reasoning task	Equal performance for TBI and HC	Functional: rest fMRI	TBI vs HC: whole brain network metrics; network connectivity within and between ROI subsystems^e^	TBI self-awareness positively associ-ated with connectivity between anterior DMN and salience network; and between posterior DMN and salience network (opposite correla-tions for controls)
Garcia-cordero et al. (2019	15 stroke	19	7/8	8/11	61.87(6.7)	67.8 (7.8)	> 6	NR	PB	Visual perception task	Less implicit monitoring in FIS than HC	Structural: MRI	Within fronto-insular stroke (FIS): whole brain voxel-based lesion-symptom mapping	Ventromedial frontal damage posi-tively associated with explicit awareness Fronto-temporo-insular damage positively associated with implicit awareness

*ABI*  Acquired Brain Injury, *ASA* Adequate Self-awareness; *AQ* Awareness questionnaire, *BI*  Barthel Index, *CT*  Computed Tomography, *DEX*  Dysexecutive Questionnaire, DMN  Default Mode Network, *DTI*  Diffusion Tensor Imaging, *DWI*  Diffusion Weighted Images, *(f)MRI*  (functional) Magnetic Resonance Imaging, *FrSBe*  Frontal Systems Behaviour Questionnaire, *FPCN*  Fronto-parietal Control Network, *ROI*  Region of Interest, *HC*  Healthy Controls, *ISA* Impaired Self-awareness, *LHD*  Left Hemisphere Damage, *NR*  Not Reported, *PCRS* Patient Competency Rating Scale, *RHD* Right Hemisphere Damage, *TBI*  Traumatic Brain Injury

^a^Injury time = time since injury in month

^b^Injury severity: S = severe, M = moderate

^c^Self-awareness assessment method: *S-P* Self-proxy Discrepancy, *PB*  Performance Based

^d^not reported but calculated based on the paper

^e^subsystems were: anterior DMN network (middle frontal, superior frontal); posterior DMN network (posterior cingulate, precuneus, temporal pole); attention network (middle temporal, lateral occipital, ventral frontal); salience network (insular cortex, anterior cingulate); frontoparietal network (precentral gyrus, superior parietal, lateral prefrontal); ‘residual’ network (sensory-motor, auditory, visual)

### Electronic Supplementary Material

Below is the link to the electronic supplementary material.

Supplementary file1 (DOCX 36 KB)

## Appendix 1: Search Terms

### Pubmed (N = 2390)

Anosognosia[Mesh] OR "Diagnostic Self Evaluation"[Mesh] OR Awareness[Mesh] OR Consciousness[Mesh] OR Metacognition[Mesh] OR denial[Mesh] OR self-perception[Mesh] OR self-concept[Mesh] OR Anosognosia*[tiab] OR "Diagnostic Self Evaluation"[tiab] OR awareness[tiab] OR consciousness[tiab] OR metacognition[tiab] OR self-regulat*[tiab] OR unaware*[tiab] OR insight[tiab] OR denial[tiab] OR self-perception[tiab] OR self-concept[tiab] OR self-appraisal[tiab] OR self-conscious*[tiab]

AND

"Brain Injuries"[Mesh] OR Stroke[Mesh] OR “brain injur*”[tiab] OR “brain trauma*”[tiab] OR “acquired brain injury”[tiab] OR stroke[tiab] OR “traumatic brain injury”[tiab] OR “brain lesion*”[tiab] OR “brain damage”[tiab] OR “cerebral injur*”[tiab] OR “cerebral trauma*”[tiab] OR “cerebral lesion*”[tiab] OR “cerebral damage”[tiab] OR “traumatic brain*”[tiab] OR “axonal injur*”[tiab]

AND

Neuroimaging[Mesh] OR “Functional Neuroimaging”[Mesh] OR “Magnetic Resonance Imaging”[Mesh] OR “Diffusion Tensor Imaging”[Mesh] OR “Diffusion Magnetic Resonance Imaging”[Mesh] OR “brain mapping”[Mesh] OR neuroimaging[tiab] OR “functional neuroimaging”[tiab] OR ("magnetic resonance"[tiab] AND (image[tiab] OR images[tiab] OR imaging[tiab])) OR “Diffusion tensor”[tiab] OR diffusion[tiab] OR connectivity[tiab] OR “brain mapping”[tiab] OR activation[tiab] OR voxel[tiab]

### Web of Science (N = 2972)

TS = (Anosognosia OR "Diagnostic Self Evaluation" OR Awareness OR Consciousness OR Metacognition OR insight OR self-appraisal OR self-conscious* OR Denial OR self-perception OR self-concept OR self-regulat* OR unaware*) OR TI = (Anosognosia OR "Diagnostic Self Evaluation" OR Awareness OR Consciousness OR Metacognition OR insight OR self-appraisal OR self-conscious* OR Denial OR self-perception OR self-concept OR self-regulat* OR unaware*)

AND

TS = ("Brain Injuries" OR stroke OR "Brain Disorders" OR "Traumatic Brain Injury" OR "Head Injuries" OR "Traumatic Brain Injury" OR "Trauma" OR "Head Injuries" OR "Brain Damage") OR TI = ("Brain Injuries" OR stroke OR "Brain Disorders" OR "Traumatic Brain Injury" OR "Head Injuries" OR "Traumatic Brain Injury" OR "Trauma" OR "Head Injuries" OR "Brain Damage")

AND

TS = ("Functional Magnetic Resonance Imaging" OR "Magnetic Resonance Imaging" OR "Neurobiological Measures" OR "Neuroimaging" OR "Brain Connectivity" OR "Diffusion Tensor Imaging" OR diffusion OR connectivity OR activation OR voxel) OR TI = ("Functional Magnetic Resonance Imaging" OR "Magnetic Resonance Imaging" OR "Neurobiological Measures" OR "Neuroimaging" OR "Brain Connectivity" OR "Diffusion Tensor Imaging" OR diffusion OR connectivity OR activation OR voxel)

### PsycINFO (N = 724)

DE Anosognosia OR DE Awareness OR DE Metacognition OR DE self-perception OR DE self-concept OR DE insight OR DE denial DE "Body Awareness" OR DE "Consciousness Disorders" OR TI Anosognosia* OR TI "Diagnostic Self Evaluation" OR TI awareness OR TI consciousness OR TI metacognition OR TI self-regulat* OR TI unaware* OR TI insight OR TI denial OR TI self-perception OR TI self-concept OR TI self-appraisal OR TI self-conscious* OR TI insight OR TI denial OR TI "Consciousness Disorders" OR AB Anosognosia* OR AB "Diagnostic Self Evaluation" OR AB awareness OR AB consciousness OR AB metacognition OR AB self-regulat* OR AB unaware* OR AB insight OR AB denial OR AB self-perception OR AB self-concept OR AB self-appraisal OR AB self-conscious* OR AB insight OR AB denial OR AB "Consciousness Disorders"

AND

DE "Brain Injuries" OR DE "Brain Disorders" OR DE "Traumatic Brain Injury" OR DE "Head Injuries" OR DE "Traumatic Brain Injury" OR DE "Trauma" OR DE "Head Injuries" OR DE "Brain Damage" OR TI "Brain Injuries" OR TI "Brain Disorders" OR TI "Traumatic Brain Injury" OR TI "Head Injuries" OR TI "Traumatic Brain Injury" OR TI "Trauma" OR TI "Head Injuries" OR TI "Brain Damage" OR AB "Brain Injuries" OR AB "Brain Disorders" OR AB "Traumatic Brain Injury" OR AB "Head Injuries" OR AB "Traumatic Brain Injury" OR AB "Trauma" OR AB "Head Injuries" OR AB "Brain Damage"

AND

DE "Functional Magnetic Resonance Imaging" OR DE "Magnetic Resonance Imaging" OR DE "Neurobiological Measures" OR DE "Neuroimaging" OR DE "Brain Connectivity" OR DE "Diffusion Tensor Imaging" OR TI "Functional Magnetic Resonance Imaging" OR TI "Magnetic Resonance Imaging" OR TI "Neurobiological Measures" OR TI "Neuroimaging" OR TI "Brain Connectivity" OR TI "Diffusion Tensor Imaging" OR AB "Functional Magnetic Resonance Imaging" OR AB "Magnetic Resonance Imaging" OR AB "Neurobiological Measures" OR AB "Neuroimaging" OR AB "Brain Connectivity" OR AB "Diffusion Tensor Imaging"

### EMBASE (N = 1843)

Anosognosia/ OR Diagnostic Self Evaluation/ OR Awareness/ OR Consciousness/ OR Metacognition/ OR insight/ OR self-appraisal/ OR Anosognosia.tw OR Diagnostic Self Evaluation.tw OR Awareness.tw OR Consciousness.tw OR Metacognition.tw OR insight.tw OR self-appraisal.tw OR self-conscious*.tw

AND

brain injury/ OR brain damage/ OR cerebral damage/ OR traumatic brain injury/ OR brain injury.tw OR brain damage.tw OR cerebral damage.tw OR traumatic brain injury.tw

AND

Functional Magnetic Resonance Imaging/ OR Magnetic Resonance Imaging/ OR Neurobiological Measures/ OR Neuroimaging/ OR Brain Connectivity/ OR Diffusion Tensor Imaging/ OR Functional Magnetic Resonance Imaging.tw OR Magnetic Resonance Imaging.tw OR Neurobiological Measures.tw OR Neuroimaging.tw OR Brain Connectivity.tw OR Diffusion Tensor Imaging.tw OR voxel.tw OR brain mapping.tw
