# Supplementary material for: Neural Correlates of Impaired Self-awareness of Deficits after Acquired Brain Injury: A Systematic Review
Source: Neuropsychol Rev. 2022 Feb 3;33(1):222–37. doi: 10.1007/s11065-022-09535-6 (PMC9998557; doi:10.1007/s11065-022-09535-6)
Supplement: Supplementary file 1 — Supplementary file1 (DOCX 36 KB) [file 11065_2022_9535_MOESM1_ESM.docx]

| Table S1. Quality assessment Tezuka et al. (2013) | | | | |
| --- | --- | --- | --- | --- |
|  | **+** | **+/-** | **-** | **Other*** |
| 1. Did they give a full description of the study participants?  (in- and exclusion criteria and patient demographics) |  | X |  |  |
| 2a. Did they give a full description of the imaging procedure and instructions? |  | X |  |  |
| 2b. Did they give a full description of the psychological task (measure of awareness) used? | X |  |  |  |
| 3. Did they specify the spatial normalization procedure, including the atlas or template which is used to match the images to? |  |  | X | NR |
| 4. Did they specify how the regions of interest were determined? |  | X |  |  |
| 5. Did they provide enough detail to reproduce the analysis? |  | X |  |  |
| 6. Are all the empirical claims supported by a specific statistical test? |  | X |  |  |
| 7. Did they describe and account for the multiple testing problem? |  |  |  | NA |
| 8. Do the figures and tables stand on their own? | X |  |  |  |
| 9. Are the quality control measures documented? |  |  | X | NR |
| Quality Rating (Good, Fair or Poor): Fair | **4.5 out of 9 🡪 5** | | | |
| *Additional Comments (If poor, please state why): 2a) Describe that they did T1/T2 weighted images, not how. 3) Not reported. 4) Not regions of interest but hemispheres. Only describe that neurologists evaluated cerebrovascular disease, but now how. 6) Not all statistics mentioned.* | | | | |
| **NA, not applicable: NR, not reported.* | | | | |

| Table S2. Quality assessment Bivona et al. (2014) | | | | |
| --- | --- | --- | --- | --- |
|  | **+** | **+/-** | **-** | **Other*** |
| 1. Did they give a full description of the study participants?  (in- and exclusion criteria and patient demographics) | X |  |  |  |
| 2a. Did they give a full description of the imaging procedure and instructions? |  |  | X |  |
| 2b. Did they give a full description of the psychological task (measure of awareness) used? | X |  |  |  |
| 3. Did they specify the spatial normalization procedure, including the atlas or template which is used to match the images to? |  |  | X |  |
| 4. Did they specify how the regions of interest were determined? |  |  | X |  |
| 5. Did they provide enough detail to reproduce the analysis? |  |  | X |  |
| 6. Are all the empirical claims supported by a specific statistical test? | X |  |  |  |
| 7. Did they describe and account for the multiple testing problem? |  |  | X | NR |
| 8. Do the figures and tables stand on their own? | X |  |  |  |
| 9. Are the quality control measures documented? |  |  | X | NR |
| Quality Rating (Good, Fair or Poor): Poor | **4 out of 10 🡪 4** | | | |
| *Additional Comments (If poor, please state why): 2a) Only mention a scan was made but not how or when. Also not mentioned how they were assessed. 3) No description of who did the neuroimaging examination or how it was done. 4) Only report regions in table 3, not how they were determined. 5) Only described very briefly. 7) Compared groups for multiple brain regions but did not correct for multiple testing.* | | | | |
| **NA, not applicable: NR, not reported* | | | | |

| Table S3. Quality assessment Lesimple et al. (2019) | | | | |
| --- | --- | --- | --- | --- |
|  | **+** | **+/-** | **-** | **Other*** |
| 1. Did they give a full description of the study participants?  (in- and exclusion criteria and patient demographics) | X |  |  |  |
| 2a. Did they give a full description of the imaging procedure and instructions? | X |  |  |  |
| 2b. Did they give a full description of the psychological task (measure of awareness) used? | X |  |  |  |
| 3. Did they specify the spatial normalization procedure, including the atlas or template which is used to match the images to? | X |  |  |  |
| 4. Did they specify how the regions of interest were determined? | X |  |  |  |
| 5. Did they provide enough detail to reproduce the analysis? | X |  |  |  |
| 6. Are all the empirical claims supported by a specific statistical test? |  | X |  |  |
| 7. Did they describe and account for the multiple testing problem? |  |  | X | NR |
| 8. Do the figures and tables stand on their own? |  | X |  |  |
| 9. Are the quality control measures documented? | X |  |  |  |
| Quality Rating (Good, Fair or Poor): Good | **8 out of 10 🡪 8** | | | |
| *Additional Comments (If poor, please state why): 6) Statistical tests done but not all statistics described. 7) Five measures of white matter integrity were correlated with awareness but no multiple testing correction reported. 8) Abbreviations not explained.* | | | | |
| **NA, not applicable: NR, not reported* | | | | |

| Table S4. Quality assessment Schmitz et al. (2006) | | | | |
| --- | --- | --- | --- | --- |
|  | **+** | **+/-** | **-** | **Other*** |
| 1. Did they give a full description of the study participants?  (in- and exclusion criteria and patient demographics) | X |  |  |  |
| 2a. Did they give a full description of the imaging procedure and instructions? | X |  |  |  |
| 2b. Did they give a full description of the psychological task (measure of awareness) used? | X |  |  |  |
| 3. Did they specify the spatial normalization procedure, including the atlas or template which is used to match the images to? | X |  |  |  |
| 4. Did they specify how the regions of interest were determined? |  |  |  | NA |
| 5. Did they provide enough detail to reproduce the analysis? | X |  |  |  |
| 6. Are all the empirical claims supported by a specific statistical test? | X |  |  |  |
| 7. Did they describe and account for the multiple testing problem? | X |  |  |  |
| 8. Do the figures and tables stand on their own? |  | X |  |  |
| 9. Are the quality control measures documented? | X |  |  |  |
| Quality Rating (Good, Fair or Poor): Good | **8.5 out of 9 🡪 9.4** | | | |
| *Additional Comments (If poor, please state why): 3+8) Table 3 mentions MNI space and talaraich coordinates, methods reports MNI. 4) Whole brain analysis.* | | | | |
| **NA, not applicable: NR, not reported* | | | | |

| Table S5. Quality assessment Ham et al. (2014) | | | | |
| --- | --- | --- | --- | --- |
|  | **+** | **+/-** | **-** | **Other*** |
| 1. Did they give a full description of the study participants?  (in- and exclusion criteria and patient demographics) | X |  |  |  |
| 2a. Did they give a full description of the imaging procedure and instructions? | X |  |  |  |
| 2b. Did they give a full description of the psychological task (measure of awareness) used? | X |  |  |  |
| 3. Did they specify the spatial normalization procedure, including the atlas or template which is used to match the images to? | X |  |  |  |
| 4. Did they specify how the regions of interest were determined? | X |  |  |  |
| 5. Did they provide enough detail to reproduce the analysis? | X |  |  |  |
| 6. Are all the empirical claims supported by a specific statistical test? |  | X |  |  |
| 7. Did they describe and account for the multiple testing problem? |  | X |  |  |
| 8. Do the figures and tables stand on their own? | X |  |  |  |
| 9. Are the quality control measures documented? | X |  |  |  |
| Quality Rating (Good, Fair or Poor): Good | **9 out of 10 🡪 9** | | | |
| *Additional Comments (If poor, please state why): 6) Not all behavioral scores have statistics. 7) Correct for multiple testing in MRI analysis. However, investigate different brain regions in ROI analysis and do not report correction for multiple testing there.* | | | | |
| **NA, not applicable: NR, not reported* | | | | |

| Table S6. Quality assessment Grossner et al. (2018) | | | | |
| --- | --- | --- | --- | --- |
|  | **+** | **+/-** | **-** | **Other*** |
| 1. Did they give a full description of the study participants?  (in- and exclusion criteria and patient demographics) | X |  |  |  |
| 2a. Did they give a full description of the imaging procedure and instructions? | X |  |  |  |
| 2b. Did they give a full description of the psychological task (measure of awareness) used? | X |  |  |  |
| 3. Did they specify the spatial normalization procedure, including the atlas or template which is used to match the images to? | X |  |  |  |
| 4. Did they specify how the regions of interest were determined? | X |  |  |  |
| 5. Did they provide enough detail to reproduce the analysis? |  | X |  |  |
| 6. Are all the empirical claims supported by a specific statistical test? |  | X |  |  |
| 7. Did they describe and account for the multiple testing problem? |  |  | X | N.R. |
| 8. Do the figures and tables stand on their own? |  | X |  |  |
| 9. Are the quality control measures documented? | X |  |  |  |
| Quality Rating (Good, Fair or Poor): Good | **7.5 out of 10 🡪 7.5** | | | |
| *Additional Comments (If poor, please state why): 7) Separately correlated orbital region, dorsolateral region, posterior region, and frontopolar regions to behavioral measure but do not correct for multiple testing. 8) Table 1 no footnotes describing what is between brackets or what abbreviations stand for.* | | | | |
| **NA, not applicable: NR, not reported* | | | | |

| Table S7. Quality assessment Grossner et al. (2019) | | | | |
| --- | --- | --- | --- | --- |
|  | **+** | **+/-** | **-** | **Other*** |
| 1. Did they give a full description of the study participants?  (in- and exclusion criteria and patient demographics) | X |  |  |  |
| 2a. Did they give a full description of the imaging procedure and instructions? | X |  |  |  |
| 2b. Did they give a full description of the psychological task (measure of awareness) used? | X |  |  |  |
| 3. Did they specify the spatial normalization procedure, including the atlas or template which is used to match the images to? | X |  |  |  |
| 4. Did they specify how the regions of interest were determined? | X |  |  |  |
| 5. Did they provide enough detail to reproduce the analysis? | X |  |  |  |
| 6. Are all the empirical claims supported by a specific statistical test? | X |  |  |  |
| 7. Did they describe and account for the multiple testing problem? |  | X |  |  |
| 8. Do the figures and tables stand on their own? | X |  |  |  |
| 9. Are the quality control measures documented? | X |  |  |  |
| Quality Rating (Good, Fair or Poor): Good | **9.5 out of 10 🡪 9.5** | | | |
| *Additional Comments (If poor, please state why): 7) Investigate inter- and intranetwork connectivity for 6 different subsystems but do not correct for multiple testing.* | | | | |
| **NA, not applicable: NR, not reported* | | | | |

| Table S8. Quality assessment Garcia-Cordero et al. (2019) | | | | |
| --- | --- | --- | --- | --- |
|  | **+** | **+/-** | **-** | **Other*** |
| 1. Did they give a full description of the study participants?  (in- and exclusion criteria and patient demographics) | X |  |  |  |
| 2a. Did they give a full description of the imaging procedure and instructions? | X |  |  |  |
| 2b. Did they give a full description of the psychological task (measure of awareness) used? | X |  |  |  |
| 3. Did they specify the spatial normalization procedure, including the atlas or template which is used to match the images to? | X |  |  |  |
| 4. Did they specify how the regions of interest were determined? |  |  |  | NA |
| 5. Did they provide enough detail to reproduce the analysis? |  | X |  |  |
| 6. Are all the empirical claims supported by a specific statistical test? | X |  |  |  |
| 7. Did they describe and account for the multiple testing problem? | X |  |  |  |
| 8. Do the figures and tables stand on their own? | X |  |  |  |
| 9. Are the quality control measures documented? | X |  |  |  |
| Quality Rating (Good, Fair or Poor): Good | **8.5 out of** **9** 🡪 **9.4** | | | |
| *Additional Comments (If poor, please state why): 4) Whole brain. 5) Smoothing not reported* | | | | |
| **NA, not applicable: NR, not reported* | | | | |
